# Apolipoprotein E ε4 accelerates the longitudinal cerebral atrophy in open access series of imaging studies-3 elders without dementia at enrollment

**DOI:** 10.3389/fnagi.2023.1158579

**Published:** 2023-05-30

**Authors:** Yuda Huang, Yongzhi Shan, Wen Qin, Guoguang Zhao

**Affiliations:** ^1^Department of Neurosurgery, Xuanwu Hospital Capital Medical University, Beijing, China; ^2^Department of Radiology, Tianjin Medical University General Hospital, Tianjin, China; ^3^Clinical Research Center for Epilepsy Capital Medical University, Beijing, China; ^4^Beijing Municipal Geriatric Medical Research Center, Beijing, China

**Keywords:** Alzheimer’s disease, cerebral atrophic, apolipoprotein E, aging, dementia

## Abstract

**Introduction:**

Early studies have reported that APOE is strongly associated with brain atrophy and cognitive decline among healthy elders and Alzheimer’s disease (AD). However, previous research has not directly outlined the modulation of APOE on the trajectory of cerebral atrophy with aging during the conversion from cognitive normal (CN) to dementia (CN2D).

**Methods:**

This study tried to elucidate this issue from a voxel-wise whole-brain perspective based on 416 qualified participants from a longitudinal OASIS-3 neuroimaging cohort. A voxel-wise linear mixed-effects model was applied for detecting cerebrum regions whose nonlinear atrophic trajectories were driven by AD conversion and to elucidate the effect of APOE variants on the cerebral atrophic trajectories during the process.

**Results:**

We found that CN2D participants had faster quadratically accelerated atrophy in bilateral hippocampi than persistent CN. Moreover, APOE ε4 carriers had faster-accelerated atrophy in the left hippocampus than ε4 noncarriers in both CN2D and persistent CN, and CN2D ε4 carriers an noncarriers presented a faster atrophic speed than CN ε4 carriers. These findings could be replicated in a sub-sample with a tough match in demographic information.

**Discussion:**

Our findings filled the gap that APOE ε4 accelerates hippocampal atrophy and the conversion from normal cognition to dementia.

## 1. Introduction

Alzheimer’s disease (AD) is a progressive neurodegenerative disease causing dementia (Scheltens et al., 2021). Apolipoprotein E (APOE) plays a significant role during the conversion from cognitive normal (CN) to AD through regulating Amyloid-β peptide (Aβ) metabolism, aggregation, and deposition (Kanekiyo et al., 2014; Mahoney-Sanchez et al., 2016; Belloy et al., 2019), in which ε4 allele of APOE is widely acknowledged as the strongest genetic risk variant for AD (Gharbi-Meliani et al., 2021; Serrano-Pozo et al., 2021).

Brain atrophy is intensely associated with the development and progression of AD (Buckner, 2004). Structural magnetic resonance imaging (sMRI) provides an accessible and non-invasive way to monitor brain atrophy with high spatial resolution and tissue differentiation (Pini et al., 2016; Rathore et al., 2017). Hence, sMRI provides a promising tool to clarify the relationships between brain atrophy and AD conversion (Jack et al., 2005), and many sMRI-based neuroimaging studies have made great contributions to AD diagnosis (Arbabshirani et al., 2017; Lian et al., 2020), as well as clinical progression prediction and monitoring (Tabatabaei-Jafari et al., 2019; Veitch et al., 2019).

APOE ε4 allele association with brain atrophy and cognitive decline has been reported in healthy aging individuals and dementia patients (Saeed et al., 2021). Moreover, cross-sectional (Burggren et al., 2008; Regy et al., 2022) and longitudinal (Lu et al., 2011; Squarzoni et al., 2018) studies demonstrated that ε4-carriers exhibit severe hippocampus atrophy during aging in CN individuals. Longitudinal studies also disclosed that ε4-carriers present greater risks of hippocampal gray matter atrophy among mild cognitive impairment (MCI) and dementia patients (Racine et al., 2018; Abushakra et al., 2020). Gray matter atrophies in several cerebral regions, including the caudate, hippocampus, and insula, were reported to correlate with APOE allele during the conversion from MCI to AD (Wei et al., 2022). However, no study has directly outlined the modulation of APOE on the trajectory of cerebral atrophy through the whole process from normal cognition to dementia. Besides, most early studies only focused on candidate brain regions, and studies illustrating the effect of APOE on cerebral atrophy from the whole brain’s voxel-wise perspective are still limited and remain to be further discussed.

Thus, this study aims to draw full trajectory maps of cerebral gray matter atrophy in the elderly with and without APOE ε4 variant from the normal cognition stage to dementia. We hypothesized that APOE ε4 would accelerate the atrophy of multiple cerebral regions during conversion from normal cognition (CN) to dementia (CN2D) and aimed to seek specific brain regions relative to AD conversion using a voxel-wise method and explored the influence of APOE variants on their atrophic trajectories. First, we investigate the effect of groups (persistent CN versus CN2D) on the gray matter atrophic trajectories with aging to seek specific target areas relative to AD conversion. Moreover, we explored the influence of APOE variants on the atrophic trajectories of target brain regions from CN to dementia stages.

## 2. Materials and methods

### 2.1. Participants

Participants enrolled in the study were acquired from the Open Access Series of Imaging Studies-3 (OASIS-3) database,^1^ which includes neuroimaging, clinical, cognitive, and biomarker data of 609 CN adults and 489 participants aged 42 to 95 years at different stages of cognitive decline. All participants were consented into Knight ADRC-related projects following procedures approved by the Institutional Review Board of Washington University School of Medicine in accordance with the Declaration of Helsinki.

**Figure 1:**
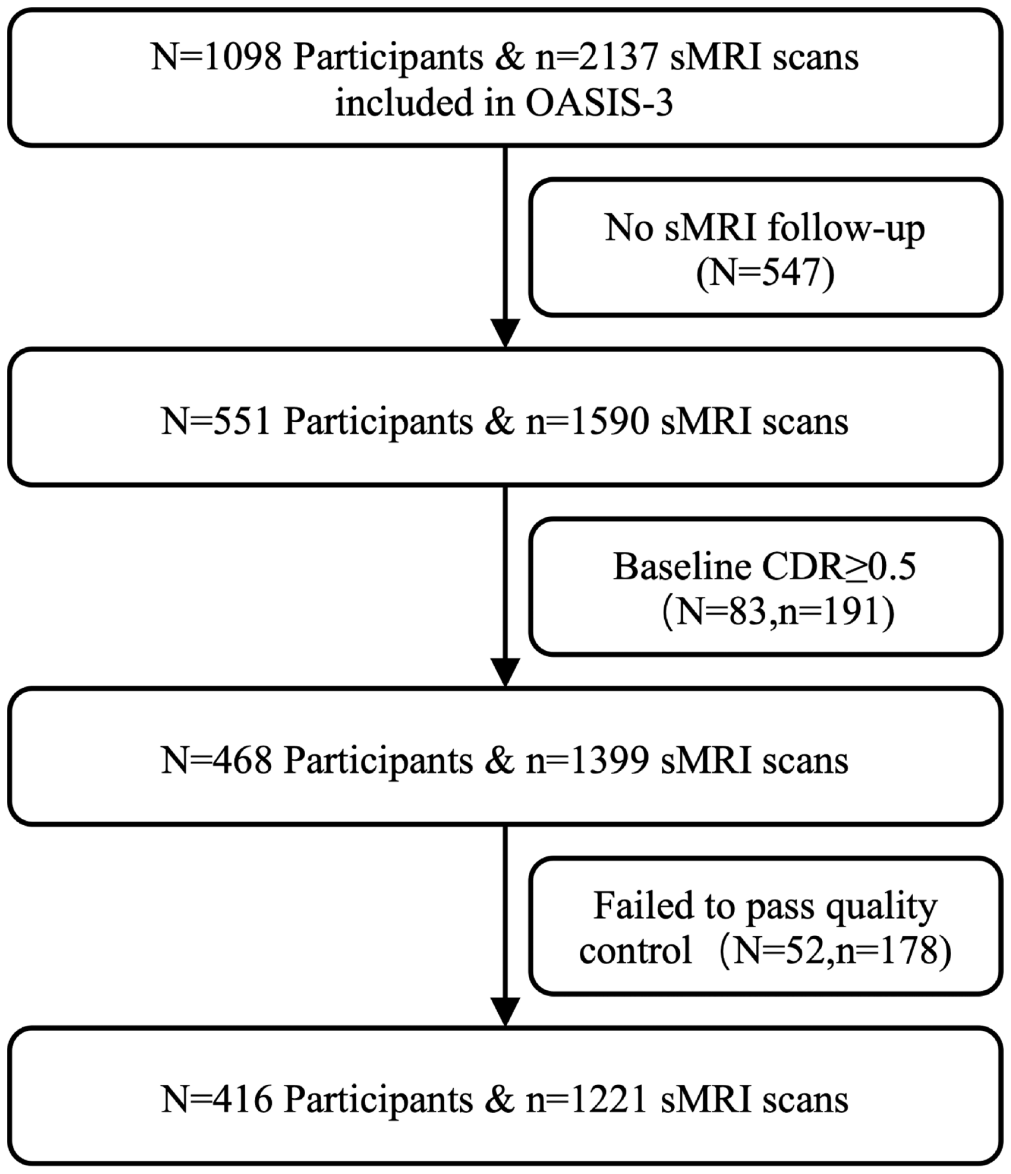
Flowchart of the study. sMRI, structural magnetic resonance imaging; CDR, Clinical Dementia Rating.

Firstly, we included subjects with at least 2 longitudinal sMRIs and corresponding clinical follow-up data, resulting in 551 participants and 1,590 sMRI scans. Secondly, we selected participants whose Clinical Dementia Rating (CDR) = 0 at baseline (removing 83 participants and 191 sMRI scans). Third, 178 sMRI scans of 52 participants were excluded because of poor imaging quality. Moreover, we categorized participants whose cognition maintained normal during all follow-up time points as the CN group and defined subjects who converted to dementia (CDR ≥ 0.5) during follow-ups as the CN2D group. Additionally, subjects carrying at least one APOE *ε*4 allele copy were defined as APOE ε4 carriers, while subjects with APOE genotype of *ε*3/*ε*3, *ε*3/*ε*2, or *ε*2/*ε*2 were recognized as APOE ε4 noncarriers. Finally, 416 subjects consisting of 54 CN2D (22 APOE ε4 carriers and 32 APOE ε4 noncarriers, 150scans) and 362 CN (107 ε4 carriers and 255 ε4 noncarriers, 1071scans) were included in the final analysis. The flowchart of participant enrollment is presented in Figure 1. Considering the mismatch in sample size and demographics between CN and CN2D groups, we additionally took CN2D subjects as the reference to match the CN participant with the same gender and age distributions.

### 2.2. Image preprocessing

The T1 weighted sMRI data of each subject were preprocessed using a longitudinal pipeline based on CAT12 toolbox^2^ for SPM12 software.^3^ Firstly, follow-up sMRI scans of every participant were rigidly co-registered to his/her baseline images. Individual-specific average T1W images were generated based on their longitudinal follow-up images. Then the average images were normalized to Montreal Neurological Institute (MNI) space using a DARTEL algorithm (Ashburner, 2007). The co-registered sMRI images of each scan were segmented into gray matter (GM), white matter (WM), and cerebrospinal fluid (CSF). Each GM map was transformed into the MNI space using the subject-specific deformation field parameters based on average images, and was modulated by the Jacobian determinants to calculate the gray matter volume (GMV). The total intracranial volume (TIV) was also generated for further statistical analysis. Finally, GMV maps were all smoothed according to an 8 mm^3^ full-width at half-maximum (FWHM) Gaussian kernel (Radua et al., 2014). Additionally, the Automated Anatomical Labeling (AAL) atlas (Tzourio-Mazoyer et al., 2002) with 90 cerebral regions was applied to extract GMV from each subjects’ sMRI scans.

### 2.3. Statistical analyzes

#### 2.3.1. Cerebral atrophic trajectories from CN to dementia conversion

In order to identify brain regions whose atrophic trajectories were specific for CN2D progression rather than consistent CN, a voxel-wise linear mixed-effects (LME) model was first introduced with GMV as the dependent variable, linear and quadratic interaction between age and group (CN2D vs. CN) as interested fixed-effects, subjects as random effects, and gender and TIV as confounding fixed-effect covariates. LME could simulate both fixed and random effects and accommodate irregular follow-up intervals with missing time nodes [Bernal-Rusiel et al., 2013; Model (1)].


(1) $$\begin{array}{l} GMV=\beta 1\;CN+\beta 2\;\left(CN\times \text{Age}\right)+\beta 3\;\left(CN\times {\text{Age}}^{2}\right)+\beta 4\;CN2D\\ \;+\beta 5\;\left(CN2D\times \text{Age}\right)+\beta 6\;\left(CN2D\times {\text{Age}}^{2}\right)+\beta 7\;TIV\\ \;+\beta 8\;\text{Gender}+\left(1+\text{Age}|\text{Subject}\right) \end{array}$$

Age and Age^2^ represent the linear and quadratic terms of age, respectively.

Intergroup differences in nonlinear GMV-age association were estimated based on joint (or multiple) hypothesis testing using the F-statistics (Shaffer, 1995; Hanck et al., 2019). Joint hypothesis testing combines multiple null hypotheses into one single test (for example, we assumed the linear and quadratic interaction terms between age and group are zero), which will return an F-statistic for this joint null hypothesis. Brain regions were regarded significant if the statistics persist after family-wise error (FWE) correction for multiple comparisons (*p* < 0.05) and cluster size larger than 20 voxels. The voxel-wise interaction between age and group was carried out using the flexible factor model design embedded in SPM12.

For brain regions showing significant age-group interaction, we extracted the mean GMV of each region of interest (ROI) to outline the atrophy trajectory as a function of age in CN and CN2D, respectively. The same post-hoc LME model (Model 1) was applied to each ROI to fit the nonlinear association between GMV and age in each group, and the joint hypothesis testing was used to test the significance of model fitting (*p* < 0.05).

#### 2.3.2. Effects of APOE ε4 on cerebral atrophic trajectories from CN to dementia conversion

To test the influence of APOE variants on longitudinal cerebral atrophy with aging, we secondly carried out another voxel-wise LME statistic with GMV as the dependent variable, linear and quadratic interaction between age and APOE variants (ε4 carriers vs. noncarriers) as interested fixed-effects, subjects as random effects, and gender and TIV as confounding fixed-effect covariates (Model 2). Similarly, a joint hypothesis testing was used to estimate the differences in nonlinear GMV-age association between the ε4 carriers and noncarriers [*p* < 0.05, voxel-wise FWE correction, with cluster-size >20 voxels; Model (2)].


(2) $$\begin{array}{l} GMV=\beta 1\;\varepsilon 4\;\text{carrier}+\beta 2\;\left(\varepsilon 4\;\text{carrier}\times \text{Age}\right)+\beta 3\;\left(\varepsilon 4\;\text{carrier}\times {\text{Age}}^{2}\right)\\ \;+\beta 4\;\varepsilon 4\;\text{non}-\text{carrier}+\beta 5\;\left(\varepsilon 4\;\text{noncarrier}\times \text{Age}\right)\\ \;+\beta 6\;\left(\varepsilon 4\;\text{noncarrier}\times {\text{Age}}^{2}\right)+\beta 7\;TIV+\beta 8\;\text{Gender}\\ \;+\left(1+\text{Age}|\text{Subject}\right) \end{array}$$

Besides, we extracted the average GMV of each ROI showing significant APOE effects. A *post hoc* analysis based on LME models was used to outline the atrophy trajectory under the effect of age in APOE ε4 carriers and noncarriers in all participants [Model (2)], and in CN and CN2D, respectively [joint hypothesis testing, *p* < 0.05; Model (3)]. A joint hypothesis test was applied to estimate the nonlinear association between GMV and age in each group and APOE variant, and to test GMV-age association differences between APOE ε4 carriers and noncarriers in each group (*p* < 0.05).


(3) $$\begin{array}{l} GMV=\beta 1\;CN\&\varepsilon 4\;carrier+\beta 2\;\left(CN\&\varepsilon 4\;carrier\times Age\right)\\ \;+\beta 3\;\left(CN\&\varepsilon 4\;carrier\times Age^{2}\right)+\beta 4\;CN\&\varepsilon 4\;noncarrier\\ \;+\beta 5\;\left(CN\&\varepsilon 4\;noncarrier\times \text{Age}\right)+\beta 6\;\left(CN\&\varepsilon 4\;noncarrier\times Age^{2}\right)\\ \;+\beta 7\;CN2D\&\varepsilon 4\;carrier+\beta 8\;\left(CN2D\&\varepsilon 4\;carrier\times Age\right)\\ \;+\beta 9\;\left(CN2D\&\varepsilon 4\;carrier\times Age^{2}\right)+\beta 10\;CN2D\&\varepsilon 4\;noncarrier\\ \begin{array}{l} \;+\beta 11\;\left(CN2D\&\varepsilon 4\;noncarrier\times Age\right)\\ \;+\beta 12\;\left(CN2D\&\varepsilon 4\;noncarrier\times Age^{2}\right)+\beta 13TIV+\beta 14\;Gender\\ \;+\;\left(1+Age|Subject\right) \end{array} \end{array}$$

#### 2.3.3. Demographic statistics

Our study analyzed demographic data using the Statistical Package for the Social Sciences version 26.0 (SPSS). Two-sample sample *t*-test (normal distribution) or Mann–Whitney U test (non-normal distribution) was used to compare differences in continuous variables between the CN and CN2D or between ε4 carriers and ε4 noncarriers (*p* < 0.05). Categorical variables, including gender and APOE status, were examined by the Chi-square test (*p* < 0.05). In addition, the consistent effect size between two separate groups was measured by calculating Cohen’s *d* value.

## 3. Results

### 3.1. Demographic characteristics

The demographic characteristics and cognitive status of all the enrolled participants are summarized in Table 1. There was no statistical difference in baseline age at the first sMRI scan (two-sample *t*-test, *t* = 1.903, *p* = 0.058), follow-up durations (Mann–Whitney *U* test, *Z* = −1.355, *p* = 0.176), number of sMRI scans per subject (Mann–Whitney *U* test, *Z* = −1.439, *p* = 0.150), baseline MMSE score (Mann–Whitney *U* test, *Z* = −0.172, *p* = 0.864), MMSE score by the end of follow-up (Mann–Whitney *U* test, *Z* = −0.606, *p* = 0.544), and gender (Chi-square test, *χ*^2^ = 0.149, *p* = 0.699) between ε4 carriers and ε4 noncarriers. CN2D patients presented a higher baseline age (two-sample *t*-test, *t* = 5.483, *p* < 0.001), worse cognitive status at the baseline (Mann–Whitney *U* test, *Z* = −2.953, *p* = 0.003), and at the end of follow-up (Mann–Whitney *U* test, *Z* = −5.625, *p* < 0.001) than CN subjects. There was no significant difference between CN and CN2D in follow-up durations (Mann–Whitney *U* test, *Z* = −0.751, *p* = 0.453), number of sMRI scans per subject (Mann–Whitney *U* test, *Z* = −1.348, *p* = 0.178). There were statistical differences between CN and CN2D in gender (Chi-square test, *χ*^2^ = 6.134, *p* = 0.013).

**Table 1:** Demographic information of all enrolled participants.

	No. Subjects	Baseline age (years)	Follow-up duration (days)	Gender (M/F)	Follow-up times	Baseline MMSE	End MMSE
ε4_Carrier	129	65.62 ± 9.27	2256.67 ± 1421.67	50/79	2.81 ± 1.14	29.03 ± 1.42	28.95 ± 1.52
ε4_non-Carrier	287	67.49 ± 9.18	2442.55 ± 1413.25	117/170	3.00 ± 1.25	29.17 ± 1.05	29.03 ± 1.81
Statistics		*t* = −1.903*	*Z* = -1.355^#^	*χ*^2^ = 0.149^@^	*Z* = -1.439^#^	*Z* = -0.172^#^	*Z* = -0.606^#^
Cohen’s *d* values		*d* = 0.203	*d* = 0.131		*d* = 0.159	*d* = 0.112	*d* = 0.048
*p* values		*p* = 0.058	*p* = 0.176	*p* = 0.699	*p* = 0.150	*p* = 0.864	*p* = 0.544
CN2D	54	73.12 ± 7.73	2224.85 ± 1201.69	30/24	2.80 ± 1.27	28.59 ± 1.60	27.52 ± 3.52
CN	362	65.98 ± 9.09	2408.78 ± 1446.05	137/225	2.96 ± 1.21	29.20 ± 1.09	29.23 ± 1.10
Statistics		*t* = 5.483*	*Z* = -0.751^#^	*χ*^2^ = 6.134^@^	*Z* = -1.348^#^	*Z* = -2.953^#^	*Z* = -5.625^#^
Cohen’s *d* values		*d* = 0.846	*d* = 0.138		*d* = 0.129	*d* = 0.446	*d* = 0.656
*p* values		*p* < 0.001	*p* = 0.453	*p* = 0.013	*p* = 0.178	*p* = 0.003	*p* < 0.001

^#^ Mann–Whitney *U* test; * Two-sample *t*-test; ^@^ Chi-square test. CN, persistent cognitive normal; CN2D, cognitive normal to dementia; sMRI, structural magnetic resonance imaging; MMSE, mini-mental state examination.

In the matched validation set, CN2D and CN participants showed no statistical difference in baseline age (two-sample *t*-test, *t* = 0.018, *p* = 0.986), follow-up durations (Mann–Whitney *U* test, *Z* = −0.771, *p* = 0.441), number of sMRI scans per subject (Mann–Whitney *U* test, *Z* = −0.157, *p* = 0.875), MMSE score at baseline (Mann–Whitney *U* test, *Z* = −0.420, *p* = 0.674), and gender (Chi-square test, *χ*^2^ = 0, *p* = 1.000), except for that CN2D had a lower MMSE score by the end of follow-up than the CN (Mann–Whitney *U* test, *Z* = −3.181, *p* = 0.001; Table 2).

**Table 2:** Demographic information of matched participants.

	No. subjects	Baseline age (years)	Follow-up duration (days)	Gender (M/F)	Follow-up times	Baseline MMSE	End MMSE
ε4_Carrier	37	71.96 ± 8.71	2231.84 ± 1575.76	22/15	2.59 ± 1.04	28.41 ± 1.95	28.05 ± 2.03
ε4_non-Carrier	71	73.71 ± 7.11	2459.73 ± 1408.91	38/33	2.83 ± 1.13	28.86 ± 1.22	28.38 ± 3.03
Statistics		*t* = −1.124*	*Z* = −1.291^#^	*χ*^2^ = 0.347^@^	*Z* = −1.164^#^	*Z* = −0.726^#^	*Z* = −1.557^#^
Cohen’s *d* values		*d* = 0.220	*d* = 0.152		*d* = 0.221	*d* = 0.278	*d* = 0.128
*p* values		*p* = 0.264	*p* = 0.197	*p* = 0.556	*p* = 0.244	*p* = 0.468	*p* = 0.120
CN2D	54	73.12 ± 7.73	2224.85 ± 1201.69	30/24	2.78 ± 1.24	28.59 ± 1.60	27.52 ± 3.52
CN	54	73.10 ± 7.74	2538.46 ± 1684.63	30/24	2.72 ± 0.96	28.81 ± 1.44	29.02 ± 1.21
Statistics		*t* = 0.018*	*Z* = −0.771^#^	*χ*^2^ = 0^@^	*Z* = −0.157^#^	*Z* = −0.420^#^	*Z* = −3.181^#^
Cohen’s *d* values		*d* = 0.003	*d* = 0.214		*d* = 0.054	*d* = 0.145	*d* = 0.570
*p* values		*p* = 0.986	*p* = 0.441	*p* = 1.000	*p* = 0.875	*p* = 0.674	*p* = 0.001

^#^ Mann–Whitney *U* test; * Two-sample *t*-test; ^@^ Chi-square test. CN, persistent cognitive normal; CN2D, cognitive normal to dementia; sMRI, structural magnetic resonance imaging; MMSE, mini-mental state examination.

### 3.2. Cerebral atrophic trajectories differences between CN2D and CN

**Figure 2:**
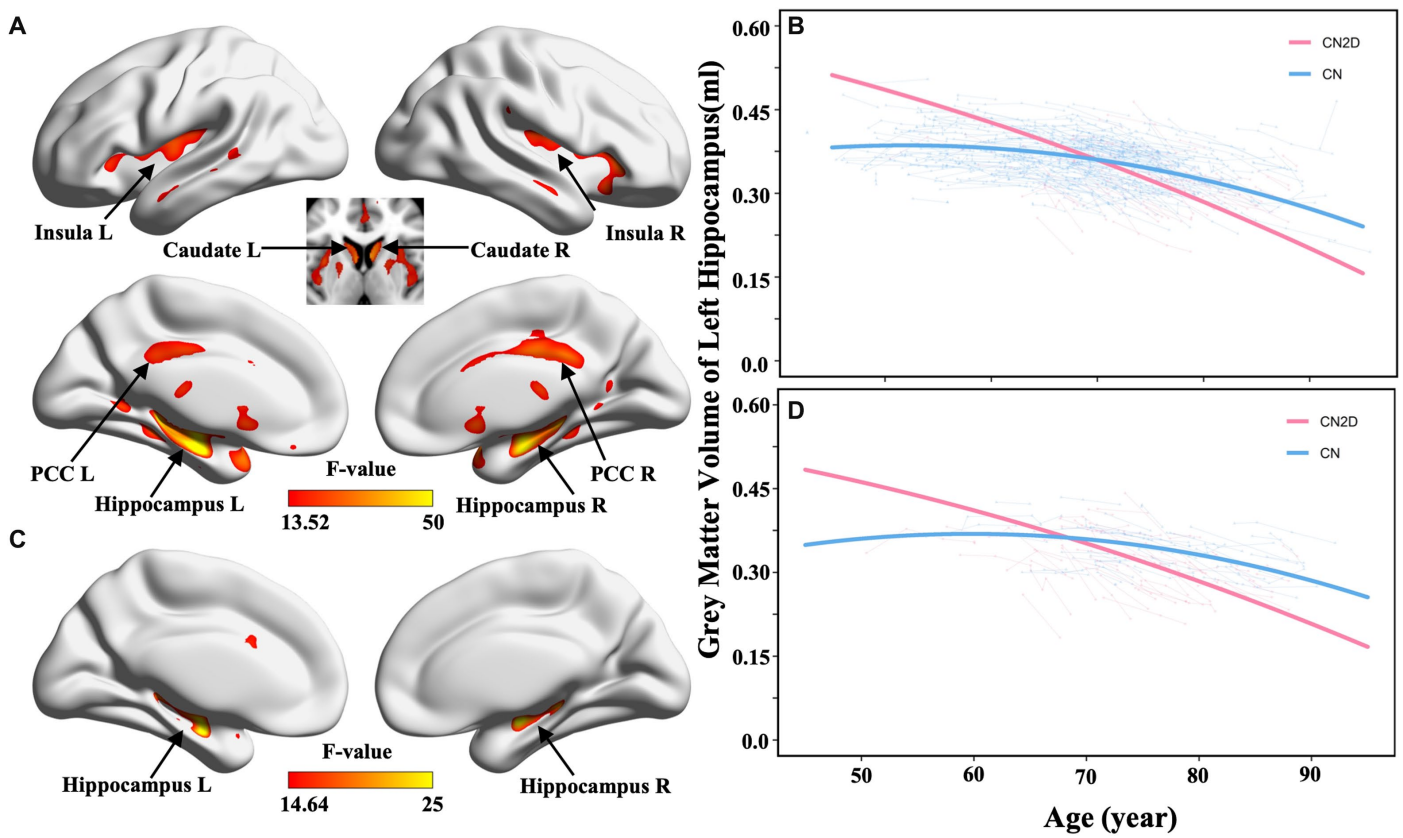
Cerebral atrophic trajectories in CN and CN2D groups. **(A,B)** represent the voxel-wise group differences in quadratic cerebral atrophy between CN and CN2D as a function of aging in the full sample dataset and validation dataset, respectively. **(C,D)** represent the ROI-wise age-related atrophy changes in the left hippocampus in the full sample dataset and validation dataset, respectively.

The voxel-wise LME model identified significant differences in nonlinear GMV atrophy following aging between all CN2D and CN participants, especially in the bilateral hippocampus, insula, post-cingulate cortex (PCC), and caudate (*p* < 0.05, voxel-wise FWE correction; Figure 2A). In the validation dataset with matched sub-samples, we also observed significant differences in GMV atrophic trajectory with aging in the bilateral hippocampi between the CN2D and CN participants (Figure 2B). ROI-wise *post hoc* analysis based on significant voxels identified by the voxel-wise LME model difference demonstrated a faster accelerated GMV atrophy in the bilateral hippocampus in CN2D than the persistent CN (left hippocampus: *F* = 60.179, *p* < 0.001; right hippocampus: *F* = 36.359, *p* < 0.001; Figure 2C; Supplementary Figure S1A), and was validated by the matched sub-sample (left hippocampus: *F* = 31.843, *p* < 0.001; right hippocampus: *F* = 21.565, *p* < 0.001; Figure 2D; Supplementary Figure S1B). For ROI-wise analysis based on AAL atlas, we similarly found bilateral hippocampus showing repeatable inter-group differences in cerebral atrophy with aging between the CN and CN2D groups (Left: *F* = 47.721, Right: *F* = 30.284, *p* < 0.05/90, Bonferroni correction). The ROI-wise result could also be validated by the matched validation sub-sample with bilateral hippocampus (Left: *F* = 17.998, Right: *F* = 20.027, *p* < 0.05/90, Bonferroni correction).

### 3.3. Effects of APOE variants on hippocampal atrophic trajectories

**Figure 3:**
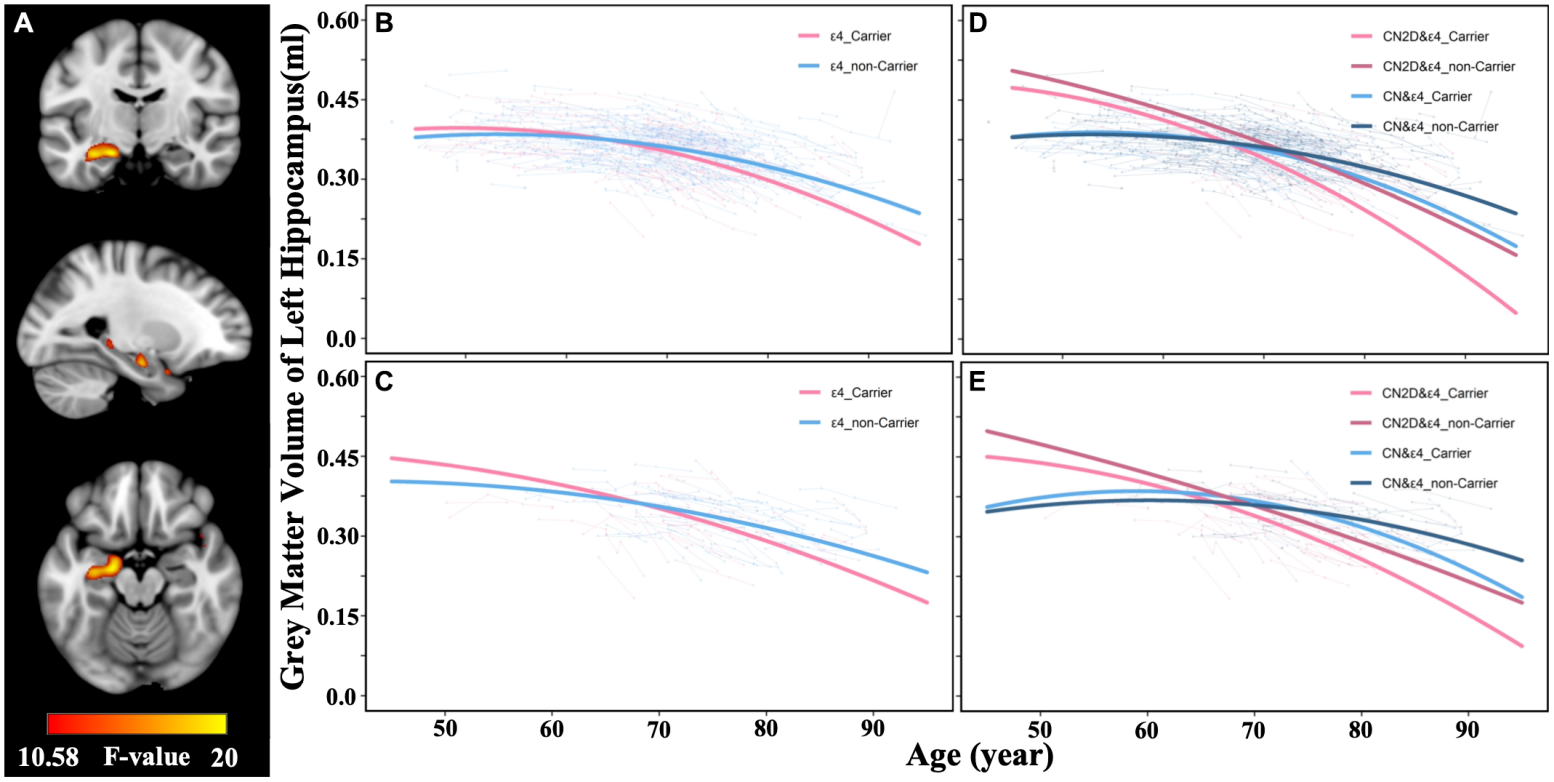
Effects of APOE variants on the cerebral longitudinal atrophic trajectories in CN and CN2D groups. **(A)** represent the voxel-wise differences in GMV atrophic trajectory as a function of aging between APOE ε4 carriers and noncarriers in the full sample dataset. **(B,C)** represent the atrophic trajectories of the identified left hippocampus in APOE ε4 carriers and noncarriers in the full sample dataset and validation dataset, respectively. **(D,E)** represent the atrophic trajectories of the left hippocampus in CN&ε4 carriers, CN&ε4 noncarriers, CN2D&ε4 carriers, CN2D&ε4 noncarriers in the full sample dataset, and validation dataset, respectively. CN, persistent cognitive normal; CN2D, cognitive normal to dementia; GMV, gray matter volume.

In the full sample dataset, the voxel-wise LME model identified a significant interaction between APOE variants (ε4 carriers vs. noncarriers) and aging on GMV atrophic trajectory in the left hippocampus (*p* < 0.05, FWE correction; Figure 3A). ROI-wise post-hoc analysis based on voxels identified significant APOE and GMV atrophy interaction showed that ε4 carriers suffered a faster accelerated quadratic GMV atrophy in the left hippocampus than the noncarriers (*F* = 15.517, *p* < 0.001; Figure 3B), which was validated by the matched sub-samples (*F* = 11.019, *p* < 0.001; Figure 3C). Similarly, ROI-wise post-hoc analysis based on AAL atlas also demonstrated a faster accelerated quadratic GMV atrophy in ε4 carriers in the left hippocampus than the noncarriers (*F* = 9.088, *p* < 0.001), which was validated by the matched sub-samples (*F* = 3.232, *p* = 0.041).

We further separated participants into four groups (CN&ε4 carriers, CN&ε4 noncarriers, CN2D&ε4 carriers, CN2D&ε4 noncarriers) according to conversion status and APOE genotype and separately outlined their left hippocampal atrophic trajectory. For voxel-wise identified ROI, we found that ε4 carriers showed a faster-accelerated atrophy than ε4 noncarriers in both CN (*F* = 8.874, *p* < 0.001) and CN2D group (*F* = 3.000, *p* = 0.050; Figure 3D), and a similar trend was validated in the validation sub-samples (CN: *F* = 2.969, *p* = 0.053; CN2D: *F* = 1.963, *p* = 0.142; Figure 3E). Meanwhile, ε4 carriers in CN2D group presented a faster atrophic speed than ε4 carriers of CN group in the full cohort (*F* = 27.8129, *p* < 0.001) and validation sub-sample (*F* = 8.899, *p* < 0.001). ε4 noncarriers in CN2D group presented a faster atrophic speed than ε4 carriers of CN group in the full cohort (*F* = 13.095, *p* < 0.001) and validation sub-sample (*F* = 3.128, *p* = 0.045; Figures 3D,E). For AAL atlas, we similarly found that ε4 carriers showed a faster-accelerated atrophy than ε4 noncarriers in both CN (*F* = 5.033, *p* = 0.007) and CN2D group (*F* = 4.962, *p* = 0.007). Meanwhile, ε4 carriers in CN2D group presented a faster atrophic speed than ε4 carriers of CN group in the full cohort (*F* = 28.982, *p* < 0.001). ε4 noncarriers in CN2D group presented a faster atrophic speed than ε4 carriers of CN group in the full cohort (*F* = 19.097, *p* < 0.001). The annual atrophic rates of the left hippocampus demonstrated an upward trend with aging, with the fastest for CN2D&ε4 carriers (0.79% at age 60, 1.81% at age 90), followed by CN2D&ε4 noncarriers (0.77% at age 60, 1.24% at age 90), CN&ε4 carriers (0.22% at age 60, 1.22% at age 90), and slowest for CN&ε4 noncarriers (0.16% at age 60, 0.83% at age 90).

## 4. Discussion

This longitudinal neuroimaging study aimed to identify cerebral regions whose atrophic trajectories contribute specifically to the transition from cognitive normal to dementia (CN2D) and outline the effect of APOE variants on cerebral atrophic trajectories during CN2D conversion. We found that CN2D participants had quadratically accelerated atrophy in bilateral hippocampi than persistent CN. Moreover, APOE ε4 carriers had faster-accelerated atrophy in the left hippocampus than ε4 noncarriers in both CN2D and persistent CN, and CN2D ε4 carriers and noncarriers presented a faster atrophic speed than CN ε4 carriers. Finally, *APOE* ε4 carriers demonstrated faster cognitive decline following aging and hippocampus atrophy. Our findings filled the gap in the mediation of APOE on brain atrophy and cognitive decline during the progression from normal cognition to dementia.

This study is based on the OASIS-3 longitudinal cohort that monitors the natural processes of aging and dementia using multimodel neuroimaging methods. Among the 416 enrolled participants that had normal cognition at the baseline, we found that about 13% (54 cases) of participants converted to dementia during an average follow-up of 6 years. Moreover, based on the data-driven voxel-based morphometry, we found CN2D participants presented faster-accelerated gray matter atrophy following aging than the persistent CN in multiple cerebral regions, especially in the bilateral hippocampi. Previous studies resembled accelerated hippocampal atrophy with aging in normal elders (Crivello et al., 2014; Fraser et al., 2015) and dementia patients (Whitwell, 2010). Moreover, faster hippocampal atrophy had also been reported in AD than in the normal elderly (Jack et al., 1998). Consistent with these studies, based on the OASIS-3 cohort, our findings highlighted the key role of bilateral hippocampal atrophy in transitioning from cognitively normal people to dementia.

APOE is widely acknowledged as a major risk gene in AD dementia (Gharbi-Meliani et al., 2021; Serrano-Pozo et al., 2021). Early studies have illustrated that APOE ε4 carriers exacerbated gray matter atrophy in the medial temporal lobe and hippocampus in patients with MCI (Schuff et al., 2009; Racine et al., 2018; Abushakra et al., 2020; Zhang et al., 2020) and AD (Li et al., 2016; Saeed et al., 2018; Bilgel and Jedynak, 2019). However, no study has paid attention to the effect of APOE on the trajectory of cerebral atrophy through the whole process from normal cognition to dementia. Our study focused on this issue by voxel-wise modeling the nonlinear atrophy trajectories of the whole brain from cognitively normal to dementia in each APOE variant. We found that APOE ε4 carriers had faster-accelerated atrophy in the left hippocampus than ε4 noncarriers in both CN2D and persistent CN. As we found significant age and gender difference of participants in the full cohort, we demonstrated a further age and gender matched validation sub-sample set in order to minimize age and gender bias. We found that APOE ε4 accelerates the longitudinal hippocampal atrophy in both full cohort and matched sub-samples. Thus, our results based on common results of exploration and validation can be regarded as eliminating the influence of age and gender. In agreement with our findings, multiple recent studies have illustrated APOE ε4 mediated accelerated cerebral atrophy in elder CN (Squarzoni et al., 2018; Gorbach et al., 2020; Van Etten et al., 2021; Regy et al., 2022). Besides, APOE ε4 also contributed to faster cerebral atrophy during the conversion from MCI to AD (Wei et al., 2022). Thus, our findings filled the gap regarding the contribution of APOE to cerebral atrophy in the transition from normal cognitive stages to dementia. Moreover, we also found that *APOE* ε4 carriers demonstrated faster cognitive decline following aging than the noncarriers, which was supported by early studies reporting APOE ε4 allele presented a significant negative modulative effect on memory, executive functioning, and overall global cognitive ability (Wisdom et al., 2011; Davies et al., 2014).

Our study suggested that bilateral hippocampus was affected during the conversion from CN to dementia while only left hippocampus suffered significant affection with APOE ε4. Asymmetric gray matter volume loss has been reported in previous study that the left hemisphere degenerates faster than the right during Alzheimer disease progression (Thompson et al., 2003). A *n* = 725 ADNI cohort analysis published by Shi rt. al. similarly found APOE ε4 carriers show significant morphological deformation difference in both CN and dementia patients affecting the left hippocampus more than the right relative to noncarriers (Shi et al., 2014). Our findings are corresponding to previous researches.

Alzheimer’s disease is characterized by the accumulation of Aβ plaques and fibral tangles containing hyperphosphorylated tau (Selkoe and Hardy, 2016). Though the mechanism between APOE and AD is still under exploration, classic neuropathological theory correlates ε4 carriers with severer Aβ plaque burden than ε4 noncarriers (Cedazo-Minguez, 2007). Animal experiments also proved that APOE deficiency mice models present decreased amyloid burden in the hippocampus (Ulrich et al., 2018). Furthermore, neuroimaging studies based on Aβ PET proved that APOE ε4 carriers suffered a higher risk of Aβ plaque in cognitively normal elders (Jansen et al., 2015) and dementia (Ossenkoppele et al., 2015). Previous studies found that the hippocampus and temporal cortex demonstrated a tight correlation between APOE ε4 mediated Aβ plaque load and cortical atrophy (Drzezga et al., 2009; Mormino et al., 2009; Chetelat et al., 2010). Besides Aβ plaque, tau pathology is being increasingly emphasized affecting AD progression. APOE ε4 carriers showed smaller volume and greater tau standardized uptake volume ratio in the hippocampus at baseline while had faster rates of atrophy and faster tau accumulation longitudinally in the hippocampus (Singh et al., 2022). An fMRI study published by Ossenkoppele et al. suggests that tau pathology drives the neurodegeneration through circumscribed brain networks in Alzheimer’s disease (Ossenkoppele et al., 2019). They similarly found that tau accumulation in lateral temporal cortex including hippocampus corresponded with cortical thickness and cognitive loss base on a tau-PET study(Ossenkoppele et al., 2019). Animal experiment indicated that the increasing Aβ plaque deposition leading to loss of large pyramidal neurons in cortical layer 5 and subiculum (Oakley et al., 2006). While microglia-mediated damage is the leading force driving neurodegeneration in a tauopathy mouse mode and further leading to gray matter volume loss (Shi et al., 2019). Thus, our findings suggested that APOE ε4 might boost the Aβ plaque deposition and tau accumulation especially in hippocampus during the full trajectory of AD progression, which further promote hippocampal atrophy and cognitive decline.

We also found that both ε4 carriers and noncarriers with CN2D conversion presented faster accelerated hippocampal atrophy than ε4 carriers with persistent CN, indicating that APOE is not the only risk gene that contributes to the progressive hippocampal atrophy during CN2D conversion. Genome-wide association studies (GWAS) have identified about 40 genetic susceptibility loci of Alzheimer’s disease, which are associated with multiple molecular pathways, such as Abeta, tau, immunity, and lipid processing (Jansen et al., 2019; Kunkle et al., 2019; Andrews et al., 2020; Wightman et al., 2021). Moreover, gene–gene and gene–environment interactions also play significant roles in AD conversion and progression (Ghebranious et al., 2011; Burke et al., 2016). Thus, these findings implied that hippocampal atrophy during the CN2D conversion is not only driven by APOE but also by other latent factors, which should be explored in the future.

Our study is not without limitations for certain. First, longitudinal follow-up sMRI data is difficult to obtain and requires the establishment of a large cohort. Although our study enrolled 416 participants in total, only 54 patients converted from CN to dementia during follow-up. Thus, the deficient sample size in CN2D patients may limit the credibility of this study. Second, due to the lack of tau PET or amyloid PET data in our longitudinal cohort, our study is not able to clarify the direct routine of how APOE allele affecting hippocampus atrophy. Further explanation needs to be combined with multi module image data. Third, our result mainly focuses on a single OASIS3 dataset. The result requires further examination with an independent database to validate its reliability.

## 5. Conclusion

Based on the OASIS-3 neuroimaging cohort, we found a faster accelerated gray matter atrophy in bilateral hippocampi during the conversion from CN to dementia than during the normal aging process. Moreover, APOE ε4 modulates a faster atrophic speed of the left hippocampus during dementia conversion. Our findings highlight the significance of APOE ε4 in accelerating hippocampal atrophy and the conversion process from CN to Dementia.

## Data availability statement

The original contributions presented in the study are included in the article/Supplementary material, further inquiries can be directed to the corresponding authors.

## Ethics statement

The studies involving human participants were reviewed and approved by Institutional Review Board of Washington University School of Medicine. The patients/participants provided their written informed consent to participate in this study. Written informed consent was obtained from the individual(s) for the publication of any potentially identifiable images or data included in this article.

## Author contributions

YH, WQ, and GZ contributed to the design and implementation of the research. YH written the first draft and revised the final draft. YS, WQ, and GZ provided valuable suggestions for the manuscript. All authors contributed to the article and approved the final version.

## Funding

This work was supported by the National Natural Science Foundation of China (82030037, 81871009, 81971599, and 81771818), STI2030-Major Projects (2021ZD0201801), the Translational and Application Project of Brain-inspired and Network Neuroscience on Brain Disorders, Beijing Municipal Health Commission (11000022T000000444685), and the Tianjin Natural Science Foundation (19JCYBJC25100). Data were provided by OASIS-3 project: Principal Investigators: T. Benzinger, D. Marcus, and J. Morris; NIH P30 AG066444, P50 AG00561, P30 NS09857781, P01 AG026276, P01 AG003991, R01 AG043434, UL1 TR000448, and R01 EB009352. AV-45 doses were provided by Avid Radiopharmaceuticals, a wholly owned subsidiary of Eli Lilly.

## Conflict of interest

The authors declare that the research was conducted in the absence of any commercial or financial relationships that could be construed as a potential conflict of interest.

## Publisher’s note

All claims expressed in this article are solely those of the authors and do not necessarily represent those of their affiliated organizations, or those of the publisher, the editors and the reviewers. Any product that may be evaluated in this article, or claim that may be made by its manufacturer, is not guaranteed or endorsed by the publisher.

## Footnotes

^1^ www.oasis-brains.org

^2^ https://neuro-jena.github.io/cat12-help/cat.html

^3^ https://www.fil.ion.ucl.ac.uk/spm/software/spm12/
