# Supplementary material for: Apolipoprotein E ε4 accelerates the longitudinal cerebral atrophy in open access series of imaging studies-3 elders without dementia at enrollment
Source: Front Aging Neurosci. 2023 May 30;15:1158579. doi: 10.3389/fnagi.2023.1158579 (PMC10265507; doi:10.3389/fnagi.2023.1158579)
Supplement: Supplementary file 1 [file Data_Sheet_1.docx]

APOE ε4 accelerates the longitudinal cerebral atrophy in OASIS-3 elders without dementia at enrollment

**Supplemental Figures:**

**
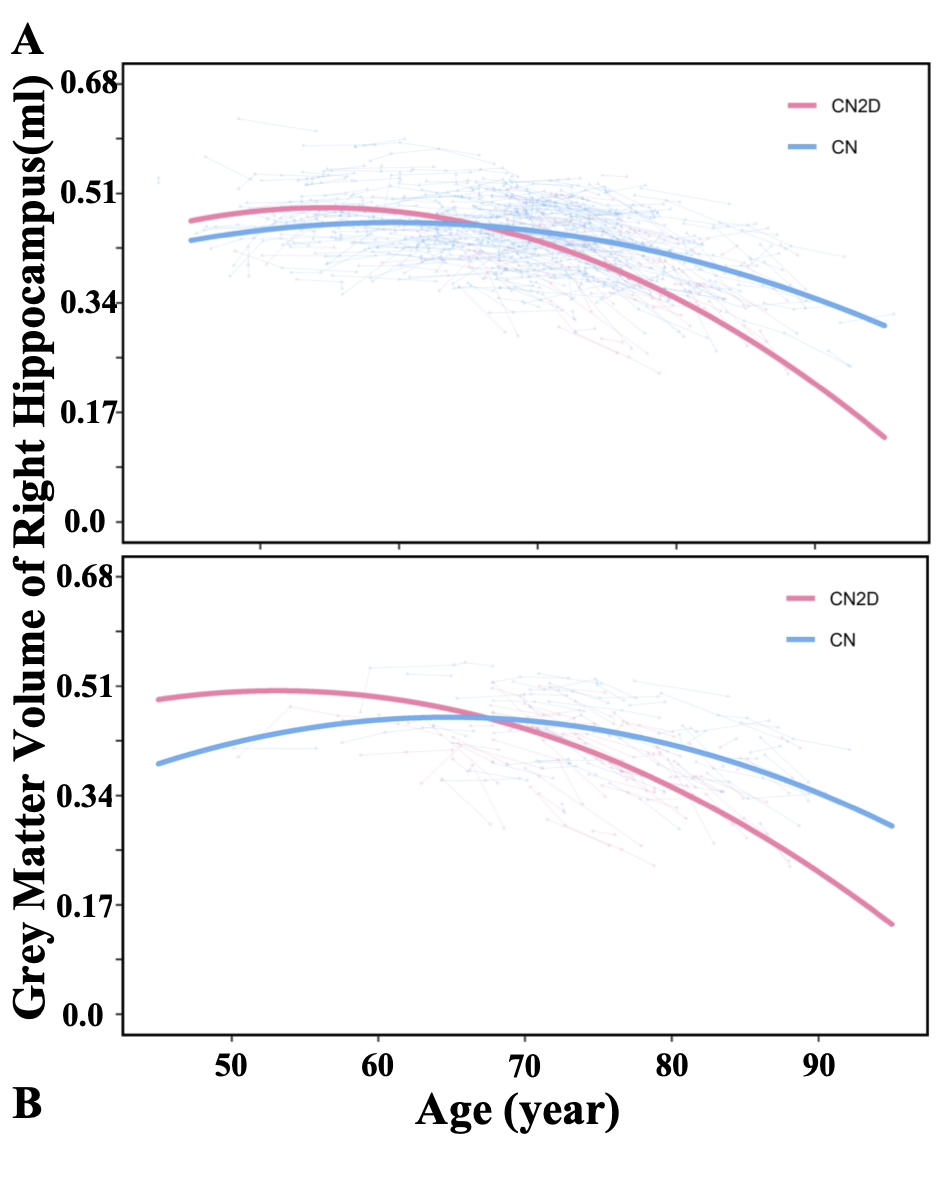
**

**Supplemental Figure 1: Cerebral atrophic trajectories in CN and CN2D groups.** (A) and (B) represent the ROI-wise age-related atrophy changes in the right hippocampus in the full sample dataset and validation dataset, respectively.


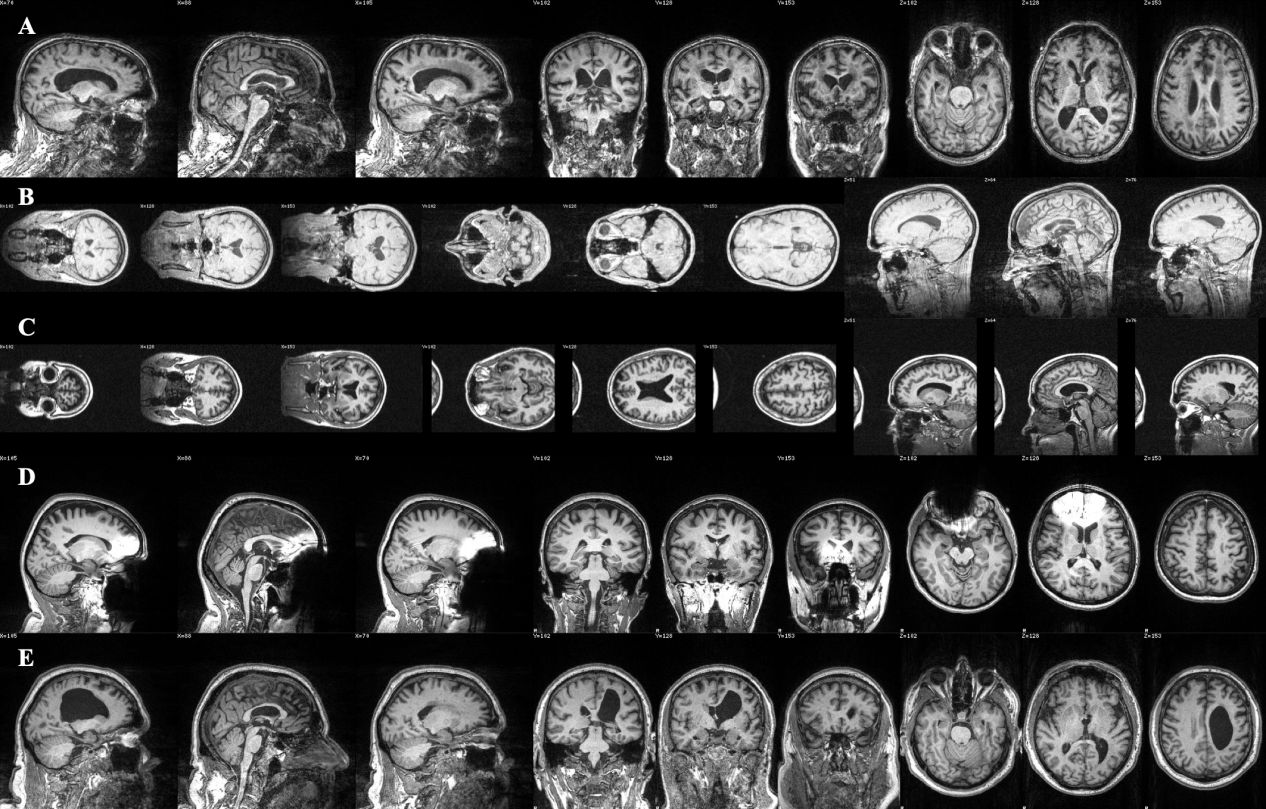


**Supplemental Figure 2: Examples of sMRI images with poor quality**

(A):head movement artifact; (B): inappropriate contrast-to-noise ratio ; (C): incomplete image scanning; (D): metal artifact; (E): ventriculomegaly.
